# Cyclopœdia of the Practice of Medicine

**Published:** 1876-07

**Authors:** 


### Bibliographical.

---

Cyclopœdia of the Practice of Medicine. Edited by Dr. H. Von Zeimssen, Professor of Clinical Medicine in Munich, Bavaria. Vol. IV., Diseases of the Respiratory Organs. Albert H. Buck, M.D., New York, editor American edition. Wm. Wood & Co., 27 Great Jones street, New York. 1876.

The fourth volume of this great work is before us. It treats of the diseases of the respiratory organs, with the affections of the nasal cavity, by Dr. Fræenkel, of Berlin; the diseases of the laryngeal mucous membrane, by Ziemssen; croup, by Steiner, of Prague; the diseases of the trachea and bronchi, by Riegel, of Wurzburg, and the diseases of the pleura by Fraentzel, of Berlin, translated respectively by G. M. Lefferts, M.D., Edward W. Schauffler, M.D., A. Brayton Ball, M.D., J. Solis Cohen, M.D., and J. Burney Yeo, M.D.

The medical profession of the United States owes a debt of gratitude to Messrs. W. Wood & Co. for the publication of this great work.

It is, according to our conviction, far the best publication of that character now in existence in the English language. Every treatise it contains is complete and finished, exhausts the subject and embraces all the advances up to date in the knowledge of the disease it treats of. It is a rational and safe guide for the practitioner, and as a book of reference in relation to the present state of knowledge existing in Germany on the etiology, morbid anatomy, diagnosis and treatment of diseases, it is unsurpassed and of incalculable value.

It will at once bring the great medical mind of this continent *en rapport* with that of Germany, and the amount of good which this publication will do to suffering mankind on the one hand, and the progress of a rational practice of medicine and medical science proper on the other, cannot be overestimated.

The work should be in the library of every intelligent American practitioner.

